# Comprehensive Sliding Wear Analysis of 3D-Printed ABS, PLA, and HIPS: ANOVA, SEM Examination, and Wear Volume Measurements with Varying Layer Thickness

**DOI:** 10.3390/polym17141899

**Published:** 2025-07-09

**Authors:** Sinan Fidan, Satılmış Ürgün, Alp Eren Şahin, Mustafa Özgür Bora, Taner Yılmaz, Mehmet İskender Özsoy

**Affiliations:** 1 Department of Airframe & Powerplant Maintenance, Faculty of Aeronautics and Astronautics, Kocaeli University, Kocaeli 41001, Türkiye; sfidan@kocaeli.edu.tr (S.F.); ozgur.bora@kocaeli.edu.tr (M.Ö.B.); 2 Department of Aviation Electrics and Electronics, Faculty of Aeronautics and Astronautics, Kocaeli University, Kocaeli 41001, Türkiye; urgun@kocaeli.edu.tr; 3 Department of Mechanical Engineering, Faculty of Engineering, Kocaeli University, Kocaeli 41380, Türkiye; alperen.sahin@kocaeli.edu.tr (A.E.Ş.); taner.yilmaz@kocaeli.edu.tr (T.Y.); 4 Department of Mechanical Engineering, Faculty of Engineering, Sakarya University, Sakarya 54050, Türkiye

**Keywords:** 3D printing, sliding wear, layer thickness, ABS, PLA, HIPS

## Abstract

This study discusses the frictional wear performance of three 3D-printed materials, acrylonitrile butadiene styrene (ABS), polylactic acid (PLA), and high-impact polystyrene (HIPS), while evaluating different layer thickness levels. The materials were subjected to wear volume and rate tests by ball-on-disc wear tests at various thickness levels (0.1, 0.2, and 0.3 mm) and sliding distances. Lastly, SEM analysis was carried out to study the wear tracks and debris developed during the testing. Quantitatively, ABS maintained a mean wear volume below 0.15 mm^3^ across all test conditions (e.g., 0.05 ± 0.01 mm^3^ at 0.1 mm layer thickness and 150 m sliding distance), whereas PLA and HIPS recorded much higher averages of 1.5 mm^3^ and 3.0 mm^3^, respectively. With the increase in layer thickness, which caused an upward trend in the obtained results, the wear volume of the investigated materials also increased. ABS exhibited the smallest material loss of all three polymers; for example, at 0.1 mm layer thickness and a 150 m sliding distance, the mean wear volume was only 0.05 mm^3^, and even under the harshest condition tested (0.3 mm layer thickness, 300 m), the value remained below 0.15 mm^3^. PLA and HIPS showed higher wear volumes, while HIPS had the lowest resistance among the three materials. The multifunctional wear behavior difference contributed by material type was 59.76%, as shown through ANOVA, and that by layer thickness was 21.32%. Among the parameters investigated, material type had the largest control in wear behavior due to inherent variation in the structural characteristics of the material such as interlayer adhesion, toughness, and brittleness. For instance, the amorphous nature of ABS and its good layer adhesion provided significantly superior wear resistance compared to the brittle PLA and the poorly adhered HIPS. It is highlighted in this research that selecting appropriate material and layer thickness combinations can improve the durability of 3D-printed components.

## 1. Introduction

Three-dimensional printing is an integrated manufacturing technology that involves several state-of-the-art fields and may probably reduce the cycle times of production and costs related to product development by an order of magnitude [1,2,3,4]. In this progress of 3D printing, several processes have been developed, and hence, a wide range of material and technique variations have started to be possible. Among them, fused deposition modeling (FDM) has become one of the most popular technologies. FDM is a type of additive manufacturing method whose process is based on the layer-by-layer manufacturing method using CAD and CAM [5,6]. Many advantages are found when using this technology: low cost of maintenance, easy change of material, no necessity for supervision, small size, and low operating temperature. In recent years, FDM-laminated parts have become increasingly common. The rise in their popularity can be attributed to their potential in the manufacturing sector to make any type of geometry. This trend is further enforced by the advanced capabilities of Finite Element (FE) software such as ANSYS (SolidWorks CAD software version 2012) and theoretical calculations, which allow for a detailed analysis of structural behaviors and key production characteristics [7,8]. Despite the advantages of FDM, the process is restricted by anisotropic mechanical behavior via layer-on-layer formation, which is prone to directional change in strength. Low adhesion between the interlayers may also reduce structural integrity, especially when in contact with a mechanical load. Porosity and rough surface are common disadvantages, which are responsible for a lack of wear resistance and dimension consistency. The disadvantages demand proper parameter optimization to optimize performance in functional applications. The anisotropic mechanical behavior of FDM parts remains a live research topic; recent high-resolution nano-indentation and surface-profilometry studies confirm that inter-filament neck growth continues to dictate friction-induced damage in ABS and PLA parts [9,10]. These analyses were performed through an appropriate combination of reliable analysis tools and a flexible production process, which allows complex and effective structures to be easily constructed. A layer is made by depositing a polymer melt-extruded filament in a pre-specified pattern, and a 3D scaffold is built by stacking these layers. Therefore, the final three-dimensional structure is dictated by the precise pattern assumed by the deposited filament within each individual layer [11,12,13,14]. In this technique, a wide range of materials can be processed, including PLA, polycarbonate, polycaprolactone, ABS, and polymeric composites [15,16]. The layer-by-layer deposition of regular filaments is one of the ways in which this process achieves the goal of building a product [9]. Friction’s effect on the wear of the engineered polymers is a consequence of microscopic and macroscopic interactions of the surfaces during relative motion [17]. Unlike the fundamental properties of materials, friction and the resulting wear cannot be represented in manuals using tabular data. Rather, because dimensional/geometrical parameters have a profound effect on the outcome, the determination of these properties requires an in-depth examination of the entire frictional contact system [18]. Wear and friction factors of polymers are as important as mechanical strength. Nowadays, considerable amounts of engineering polymers are required for several couplings, sliding doors, and guide rails. In such cases, they often encounter direct contact with other materials, metals, and plastics. Therefore, for the appropriate evaluation of friction and wear, a thorough understanding of the system conditions is required [19].

One factor, among the main and controllable factors affecting the physical properties of 3D-printed materials, is the thickness of the printing layer [20]. Wu et al. [21] studied the changes in the mechanical properties of 3-D-printed PEEKs with changing raster angles and layer thicknesses. They fabricated samples using a PEEK 3D printing machine with three different layer thicknesses and raster angles, specifically 200, 300, and 400 µm, and 0°, 30°, and 45°, respectively. Then, tensile, compressive, and bending strength tests were conducted on the samples. Their test results showed that the best mechanical properties could be obtained for a 0° raster angle and 300 µm layer thickness. While PEEK is different in thermal and mechanical properties than ABS, PLA, and HIPS, our findings highlight the primacy of optimizing layer thickness—a parameter of particular concern herein. This observation warrants our detailed study of the effects of layer thickness on wear resistance in typical FDM polymers. In the same way, Farzadi et al. [22] investigated how four different layer thicknesses and three different printing orientations parallel to X, Y, and Z affected the toughness, Young’s modulus, and compressive strength of the scaffolds. The results revealed that the toughness, compressive strength, and Young’s modulus in samples made using the 0.125 and 0.1125 mm layer thicknesses had better quality than those of the other samples. SEM and mCT tests indicated that the size accuracy, porosity, and the ability for pores to connect with each other in samples printed in X directions in a 0.1125 mm thick layer were higher than those in samples prepared using the SolidWorks CAD software of version 2012. Hardness, friction, and wear tests showed that applying thickness and orientation factors in 3D printing can influence polymer behavior in different ways. Under a normal load of 100 N, the layer thickness varied with the wear rate, whereas the influence of the layer orientation remained unclear. Based on this study, the layer orientation and thickness should be selected based on each design problem. Additionally, because of the nature of 3D printing, different production parameters may have varied effects on the qualities of distinct polymer parts [23]. Three distinct print orientations and filament colors were used to extensively explore the tribological behavior of FDM 3D-printed PLA. Tribological tests were conducted using two applied loads. Surface roughness (Ra) values for all specimens decreased significantly during testing, ranging from 75.37% to 85.21%, compared with the virgin surface. This notable decline highlights the impact of print orientation and filament color on the wear properties of PLA under applied loads and validates the presence of wear, mainly due to the removal of rough outer layers during sliding contact [24]. The mechanical properties were characterized in this study by changing the 3D printing layer thickness and post-printing techniques of a dental resin material. Accordingly, the results showed that applying a 100 μm layer thickness produced the highest flexural strength compared to 25 and 50 μm. Notably, the flexural strengths of all the groups evaluated were above the minimal 50 MPa criterion for temporary crown materials [25]. Wear characteristics are highly affected by the layer thickness. The thinner the layers, the less wear there is; out of all the infill options, the grid pattern showed the least wear. In addition, it has been proven that decreasing the layer thickness and increasing the infill density improve the wear resistance. Generally, testing results indicate that the build orientation, layer thickness, and material choice significantly affect the wear behavior and, consequently, the potential load-carrying capacity of 3D-printed parts [26]. Instead, the following polymeric substitute mouthguard materials are introduced: thermoplastic polyurethane, high-impact polystyrene, poly (methyl methacrylate), and recycled poly (lactic acid). Although the study provides enlightening data on the mechanical, chemical, thermal, and surface properties of such materials, further study is needed to clearly explain the trend towards reduced impact strength with increasing thickness. Although TPU is an exception to this general trend, its individual deformation capability is worthy of more detailed analysis [27]. Norani et al.’s study of FDM parameters shows mechanical properties are greatly affected by layer thickness in terms of interlayer adhesion and porosity. Lower layer thicknesses enhance bond strength and diminish voids, which in turn, impacts wear resistance. This correlation necessitates emphasis on layer thickness as the key parameter for exploring wear performance in ABS, PLA, and HIPS, although in recognition of the secondary effect of the other parameters in material behavior [28]. While these parameters result in low adhesion, elevated plastic deformation, buckling, and weak interlayering, they also improve the thermal bonding and amplify the mechanical strength. The porosity is anisotropic in 3D-printed polymers, although their elastic properties balance their absorption capability. Under certain sliding conditions, asperity break, increased contact area, and plastic deformation lead to low friction and wear, which improves their performance in wear-critical applications. Popescu et al. [29] investigated how different processing parameters strongly influence the mechanical behavior of thermoplastic materials fabricated by FDM. The mechanical properties of printed parts are highly influenced by critical parameters such as build orientation, layer thickness, air space, infill density, and raster orientation. These results justify the optimization of these parameters to enhance the strength, durability, and functionality of thermoplastic 3D-printed structures, providing valuable insights for future research and applications in additive manufacturing. Pant et al. [30] investigated the influence of significant FDM process variables on PLA material wear rate. The results indicated, with regard to experiment design, that orientation was the most important component, with a *p*-value of 0.009. A layer thickness of 0.3 mm, orientation of 90°, and extruder temperature of 220 °C were found to be the ideal operating parameters. This resulted in a minimum wear rate of 456.43, which is important information for wear-critical applications. Türksayar and Diker [31] used the V-Print splint resin in a 3D printer with digital light processing technology; specimens with three different layer thicknesses (50, 75, and 100 µm) were made, leading to the creation of discs that were 3 mm thick. For the two-body wear tests, the specimens were placed through 120,000 cycles of a chewing simulator. Layer thicknesses of 50 µm and above had no effect on the material’s wear resistance, according to statistical analysis using the Shapiro–Wilk test, two-way ANOVA, one-way ANOVA, and Tukey post hoc test (α = 0.05). Consequently, for faster printing without sacrificing wear performance, a layer thickness of 100 µm is recommended.

This paper makes a significant contribution to this field of research by using ANOVA, SEM, and wear volume measurements to investigate the sliding wear on 3D-printed ABS, PLA, and HIPS materials that are made of different thicknesses. The present study extends the existing knowledge about the sliding behavior of different types of 3D-printed polymers by systematically investigating the influence of the layer thickness on wear parameters. The present work bridges the existing literature gap by integrating statistical techniques with an extensive microscopic investigation to establish the ranking in wear resistance of these widely used additive manufacturing materials. The outcomes of this study will enhance the design of material selection and optimization methodologies for applications that require high wear resistance, and will significantly contribute to the development of more dependable and reliable 3D-printed components.

## 2. Materials and Methods

### 2.1. Materials

In the present study, the test specimens were prepared using three types of 3D printing filaments: PLA, ABS, and HIPS. The design was made using Autodesk Fusion 360 of version 2022 software and was kept constant for all samples, with dimensions of 40 × 40 × 3 mm. Samples of each material type were printed with three different layer thicknesses to investigate the sliding wear behavior variation with respect to the layer thickness.

The CAD models designed in Fusion 360 were all exported in STL format for further processing. The STL files were then imported into Cura 5.0 slicing software, which required slicing parameters to be changed with respect to material and layer thickness for each print. The specimens were fabricated on an ANYCUBIC MEGA S fused deposition modeling (FDM) printer (ANYCUBIC, Shenzhen, China) equipped with a 0.40 mm brass MK8 nozzle and a 1.75 mm filament drive. Printing was performed at a constant toolhead travel speed of 50 mm s^−1^ (extrusion speed 25 mm s^−1^), with nozzle temperatures of 210 ± 2 °C (PLA), 240 ± 2 °C (ABS), and 230 ± 2 °C (HIPS); bed temperatures of 60 °C, 100 °C and 70 °C, respectively; and a build-plate fan duty cycle of 100% (PLA) or 50% (ABS, HIPS). All parts were printed at 100 % rectilinear infill, a 45° raster angle, and the three target layer heights (0.10, 0.20, 0.30 mm); chamber conditions were maintained at 23 ± 2 °C and 50 ± 5% RH to minimize environmental variability. For each configuration of material–layer thickness, the layer deposition was carried out consistently and accurately. All the specimens were fabricated under controlled conditions to ensure reliable analysis of their wear characteristics during subsequent testing.

**Figure 1:**
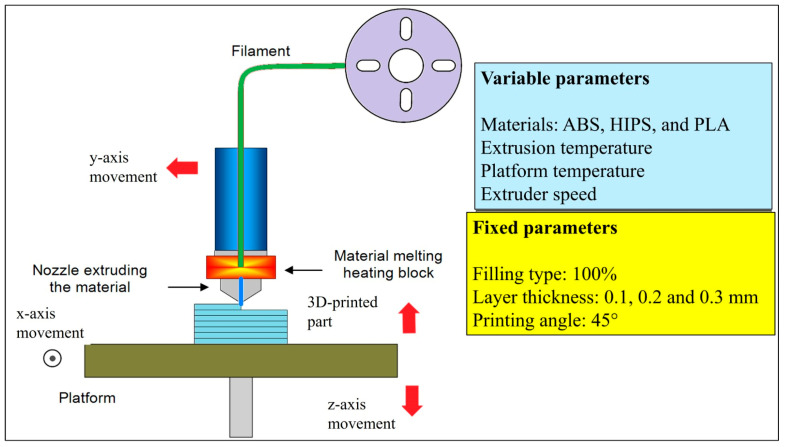
Manufacturing process using a 3D FDM printer.

Figure 1 illustrates the fundamental stages of the FDM (fused deposition modeling) 3D printing process, together with the associated variable and fixed parameters. This is particularly useful for analyzing friction wear in 3D printers composed of materials like ABS (acrylonitrile butadiene styrene), PLA (polylactic acid), and HIPS (high-impact polystyrene). The parameters in the blue box in the figure below are those that can be changed throughout the printing process. Such variables include the material used for printing (for example, ABS, PLA, HIPS), extrusion temperature, platform temperature, and extruder speed, which all affect the quality of the print and, therefore, the mechanical properties of the printed components. For instance, ABS exhibited a higher melting point than PLA and superior impact resistance. The platform temperature influences the material’s adhesion to the build surface and the diffusion between successive raster’s, as higher bed temperatures slow the cooling rate, enhance polymer chain mobility, and thus strengthen weld lines [13,32]. This, in turn, directly governs surface quality and downstream wear performance. The extruder speed regulates the material output velocity and ensures an accurate arrangement of the layers in accordance with the printing duration. The fixed parameters used in the experimental investigation are the standardized variables indicated in the yellow box. The settings include a 100% fill rate, layer thicknesses of 0.1, 0.2, and 0.3 mm, and a print angle of 45°. A value of 100% means that all printed components are completely solid, which is one of the most influential factors on durability during wear testing. Layer thickness is another factor considered very influential as it directly affects the surface roughness and wear resistance. A thin layer normally results in a much finer surface, whereas thicker layers increase the roughness of the resulting surface and hence increase the wear volume. The manufacturing angle is considered an important factor that influences the anisotropy of the mechanical properties of a component, especially the wear behavior and bonding between layers. The materials used in wear analysis, layer thickness, and printing parameters are critically important. The materials used in this study exhibit different wear characteristics in relation to their mechanical properties. ABS is known for its toughness and resilience, whereas PLA is biodegradable but brittle. HIPS offers superior impact resistance. The influence of these parameters on wear volume was statistically assessed using ANOVA (analysis of variance), while SEM (scanning electron microscopy) analysis was utilized to examine the surface morphology after wear.

### 2.2. Sliding Wear Test and Wear Volume Calculation

**Figure 2:**
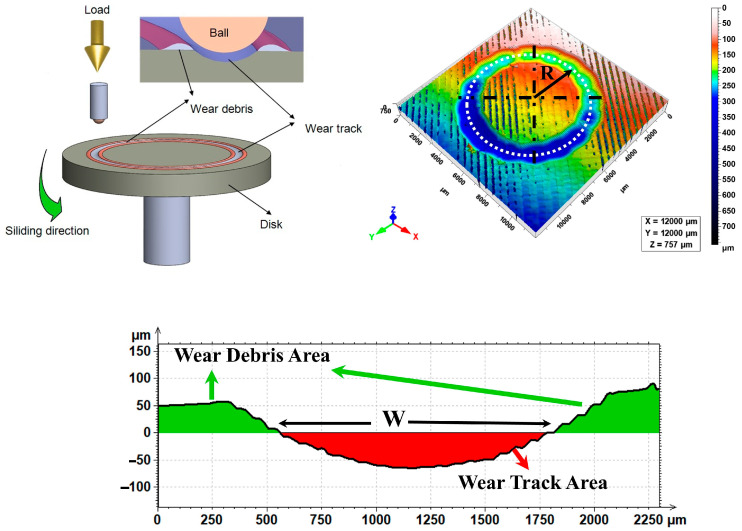
Ball-on-disc wear test and wear volume calculation steps from the wear track and wear debris areas.

The sliding wear tests were conducted on the Nanovea T50 tribometer (Nanovea, Irvine, CA, USA) in accordance with the ball-on-disc test method of the ASTM G99 test standard. The test specimens were run under 20 N of normal load and 1 m/s rotational speed, 4 mm rotational radius, and for 150 m and 300 m distances, respectively, in ambient humidity and temperature. The ball-on-disc wear test, which is displayed in Figure 2, is the test employed to examine the 3D-printed material’s wear behavior. The ball in a stationary state and the rotating disc in a constant-load state are displayed in the schematics of the wear-test rig below. The rotating disc, which is the material sample, rotates in a clockwise direction and the ball rubs on the rotating disc, creating on the surface of the disc the image of the wear track. The wear volume is then achieved through the analysis of the profile and the wear debris.

The wear-track geometry was measured using a The Nanovea PS-50 non-contact laser 3D surface profilometer (Nanovea, Irvine, CA, USA). Based on the results, a detailed topographic map of the worn surface was obtained. The profilometric data indicated two areas on the surface: the first is the wear track from where material was removed, and the other is the wear debris area, being the place where the displaced material resides.

The wear volume (W) was calculated from the profile of the wear track by integrating the cross-sectional area of the wear track along the wear path using Mountains Technology DigitalSurf Software (version 6.2.7487). The profilometer data provide the depth and width of the wear track, thereby enabling an exact calculation of the wear volume. This method allows an accurate assessment of material loss and debris formation—information important to a comparative study on wear resistance between different 3D-printed materials under specific conditions. For each combination of material (ABS, PLA, HIPS), layer thickness (0.1, 0.2, 0.3 mm), and sliding distance (150 m and 300 m), three replicate specimens were made and subject to tests of sliding wear. The use of the triplicate test method here served to replicate the results and augment the statistical reliability. When comparing the wear marks, the means of three repetitions of each test were used to establish the results and the percentage error margins are displayed in the respective graphs.

The calculation of the Wear Rate, which quantifies the rate of change in wear volume, was conducted in accordance with Formula (1) (as specified by the ASTM G99 test standard), where *k* is the wear rate (mm^3^/N.m), *V* is the wear volume (mm^3^), *L* is the normal load (N) and *X* is the sliding distance (m).

(1) $$k=\frac{V}{L*X}$$

This integrated approach ensures that the measured wear data are reliable and reproducible, which is critical for comparing the ABS, PLA, and HIPS wear performance under varying layer thicknesses and sliding distances. The methodology is in line with standard testing protocols for tribology and provides a stable platform for understanding wear behavior in 3D-printed materials.

### 2.3. SEM Imaging

A JEOL scanning electron microscopy (SEM) (JEOL, Tokyo, Japan) machine and SEM photographs were used to examine in detail the wear mechanisms and surface morphology of the composite samples. Scanning electron microscopy analysis was conducted using a JEOL SEM instrument (JEOL) operated at 15 kV accelerating voltage. Micrographs were captured at 30× magnifications. The secondary electron imaging mode was employed to examine the surface topography of wear tracks and debris. These standardized conditions ensured consistent imaging quality and repeatability of observations across all material specimens. The polymer specimens were further gold (Au)-coated prior to observation through scanning electron microscopy (SEM). This procedure was carried out in order to achieve better image resolution and to avoid the accumulation of electrical charges on the sample surface. Sputter coating of the specimens was performed in a vacuum state of 10^−2^ mbar, with a current of 30 mA and for 120 s of coating time. The parameters thus generated the deposition of a gold coating of 1–2 µm mean thickness across the surface of the material.

## 3. Results and Discussions

### 3.1. Wear Track Area and Wear Debris Area Analysis

**Figure 3:**
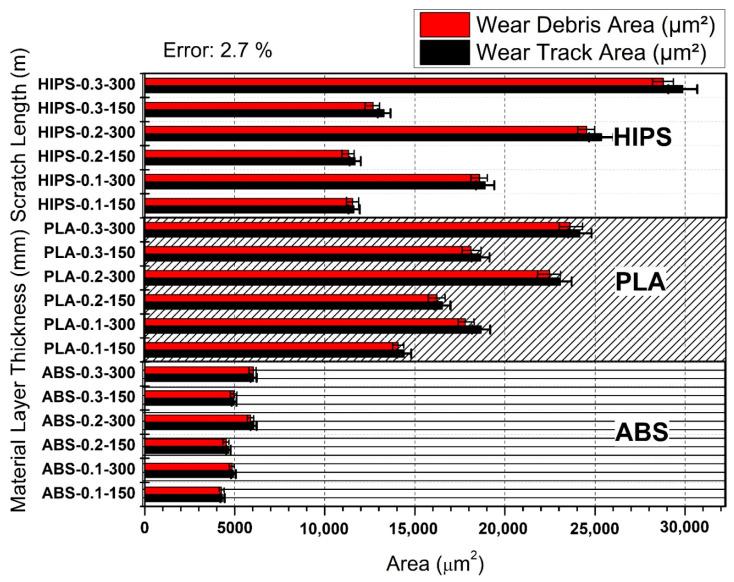
Comparison of wear debris and wear track areas in 3D-printed HIPS, PLA, and ABS samples with various layer thicknesses (Error: 2.7%).

Figure 3 presents a detailed comparative analysis of the wear debris area versus wear track area for 3D-printed samples of three different materials—HIPS (high-impact polystyrene), PLA (polylactic acid), and ABS (acrylonitrile butadiene styrene)—regarding their wear resistance. The analysis was conducted in relation to varying thicknesses of the layers and lengths of the scratches, two important factors that influence the wear resistance of these materials. The results provide insight into how the mentioned parameters influence the wear properties of the materials. In addition, from Figure 3, it can be observed that the wear debris area as well as the wear track area increased with the layer thickness and sliding distance in HIPS. For example, a layer thickness of 0.3 mm on HIPS and a sliding distance of 300 m led to the highest wear debris area that can be expected, thus showing a higher volume of material lost from the surface. On the other hand, HIPS with a layer thickness of 0.1 mm and sliding distance of 150 m exhibited the lowest wear debris and wear track areas. A similar trend of increased wear debris and wear track area with increasing layer thickness and scratch length was observed with the PLA section. However, the magnitude of wear exhibited by PLA was less than that of HIPS, especially at lower layer thicknesses and for shorter sliding distances. For example, medium wear was recorded for a 0.2 mm thick PLA layer with a sliding distance of 150 μm, meaning that under some settings, PLA can be a trade-off between the rigidity of the structure and wear resistance. In the ABS section, the wear debris and wear track areas were much lower than those for HIPS and PLA under all test conditions. These results suggest that ABS exhibits far superior wear resistance, and with the same high-impact strength, it could be quite a good material for applications that demand high wear resistance. In particular, ABS with a layer thickness of 0.1 and a sliding distance of 150 m showed the minimum wear debris area, corresponding to better performance under these conditions.

Figure 3 shows the extent to which the wear damage mechanism occurs as plastic deformation and/or material loss during the wear test. In the wear test results, the difference between the trace area formed on the sample surface and the debris area represents the area of material lost from the sample surface, whereas the debris area represents the wear trace damaged by plastic deformation [33,34,35,36,37,38]. In Figure 3, the wear mechanism changes from plastic deformation to material loss as the sliding distance increases for all three material types. When the effect of polymer type on the wear mechanism is examined, it is clearly observed that the damage occurring in ABS is in the form of a plastic deformation. It can be seen that the wear track and debris areas are approximately equal. Thus, the wear damage remained at the level of plastic deformation on the material surface. Considering the PLA and HIPS test results, it can be seen that, unlike for ABS, damage occurs on the surface not only in the form of plastic deformation but also as material loss. As the layer thickness increased, the material loss rate on the sample surface increased. This increase proves that, as the layer thickness increases, the interlayer gap increases and adhesion weakens. Consistent with this interpretation, Liu et al. [20] showed that reducing the printing layer to 0.10 mm lowered the coefficient of friction by 18% compared with 0.30 mm layers, owing to denser interfacial bonding. Likewise, Hanon et al. [24] reported a 25% decline in wear-scar width for PLA and ABS when optimized layer settings minimized the dynamic friction coefficient.

As stated in ref. [39], for both 3D-printed PLA-PCU and PLA materials, the results show a correlation between a higher applied normal load and thicker printing layers, with a lower coefficient of friction (COF) and wear rate. The progression of asperity deformation under these conditions is illustrated by the micrographs of the worn surfaces. Interestingly, the surface deformation increased with increasing weight. This is primarily due to the thicker printing layer (0.16 mm) compacting the overlapping raster layers. This indicates that the tribological behavior of 3D-printed polymers is strongly influenced by the load and layer thickness. According to ref. [40], the volume loss values of the PLA and PLA/CF components fluctuated in line with changes in the infill rate and duration of water absorption. It was found that the volume loss values decreased as the infill rate increased from 20% to 100%. The coefficient of friction (COF) values, which varied from 0.37 to 0.75 for both the PLA and PLA/CF parts, showed a similar pattern. The results of this study indicate that enhancing the infill rate mitigates not only material loss but also influences the frictional behavior of the components, thereby enhancing the resistance to wear at elevated rates. In ref. [41], the exceptional wear resistances of 3D-printed polymers (ABS, PLA, Co-polyester, and PCL) were reported. These are explained by the improved bonding strength made possible by 3D printing technology. Reducing the layer thickness is essential for increasing the bonding strength of FDM-printed items. As the layer thickness decreases, the bonding strength increases. The gap between the contour and raster in all print orientations was minimized when the layer thickness was 125 µm (0.1 mm) in this investigation, which may have contributed to the printed specimens’ overall mechanical performance and excellent wear resistance. Kovan et al. [42] quantified the effect of layer thickness and print orientation on the adhesive strength of 3D-printed parts. It was observed that, at a lower layer thickness, the edgewise orientation possesses the maximum bonding strength, whereas at a higher layer thickness, the flatwise orientation has the maximum bonding strength. Similarly, in the case of adhesion strength, the edgewise orientation outperformed the flatwise orientation at greater layer thicknesses. On the other hand, the adhesion strength was superior for edgewise orientation at low layer thickness. These results highlight the urgent need for improvements in the bonding and adhesive characteristics of 3D-printed parts with regard to print orientation and layer thickness. Dangnan et al. [43] reported that for both 3D-printed polymers, the surface orientation during sliding affected wear resistance. The result shows that in 3D-printed ABS, in which the test specimens were oriented perpendicular to the sliding direction, the specific wear rate decreased; this means that surface abrasion was less pronounced in the 3D-printed ABS than in the parallel orientation.

Therefore, Figure 3 provides important knowledge regarding the wear performance of 3D-printed materials, pointing out the effect of the layer thickness and scratch length on the wear properties. The results of this research indicate that both HIPS and PLA exhibit higher wear rates under increased stress conditions, whereas ABS exhibits better wear resistance in all cases, making it a preferred choice for high-strength durability applications. Such observations are crucial for optimizing 3D printing parameters and thus improving the lifespan and performance of printed components.

### 3.2. Wear Volume and Wear Rate Analysis

**Figure 4:**
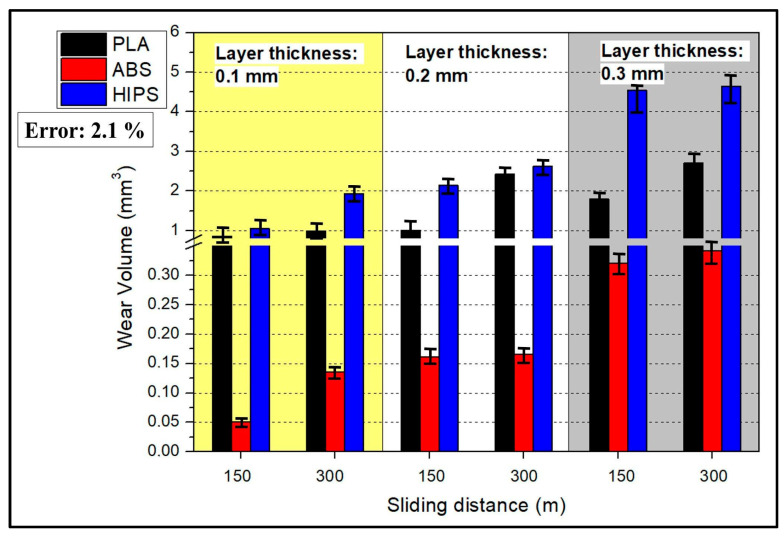
Wear volume results (Error: 2.1%).

The wear volume results of the wear marks formed on the sample surfaces as a result of the wear test are shown in Figure 4. When the results were evaluated, it was clearly observed that ABS exhibited the highest abrasion resistance, whereas HIPS was the weakest polymer. When the effect of layer thickness on wear resistance was examined, the amount of wear on all three polymers was found to increase with increasing layer thickness. Thus, it can be clearly observed that the increase in layer thickness during sample production with a 3D printer negatively affects wear resistance. The increase in the friction distance, which was used as the test parameter, increased the wear damage to the sample surfaces, as expected.

**Figure 5:**
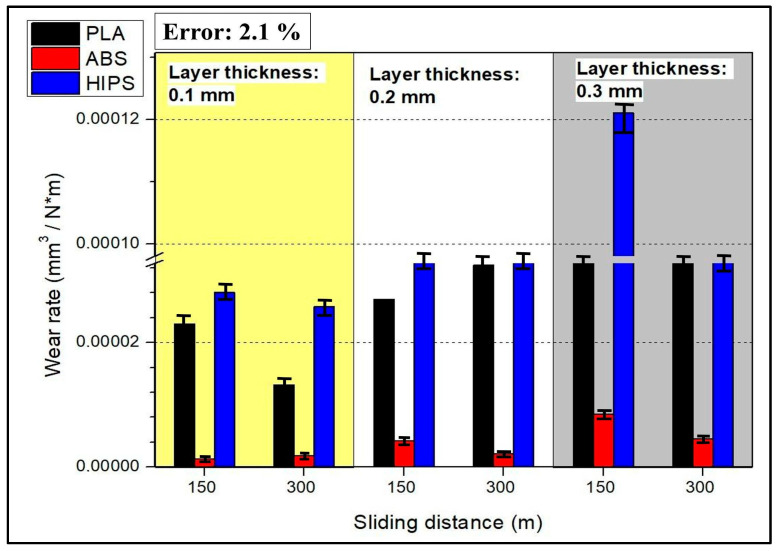
Wear rate results (Error: 2.1%).

**Figure 6:**
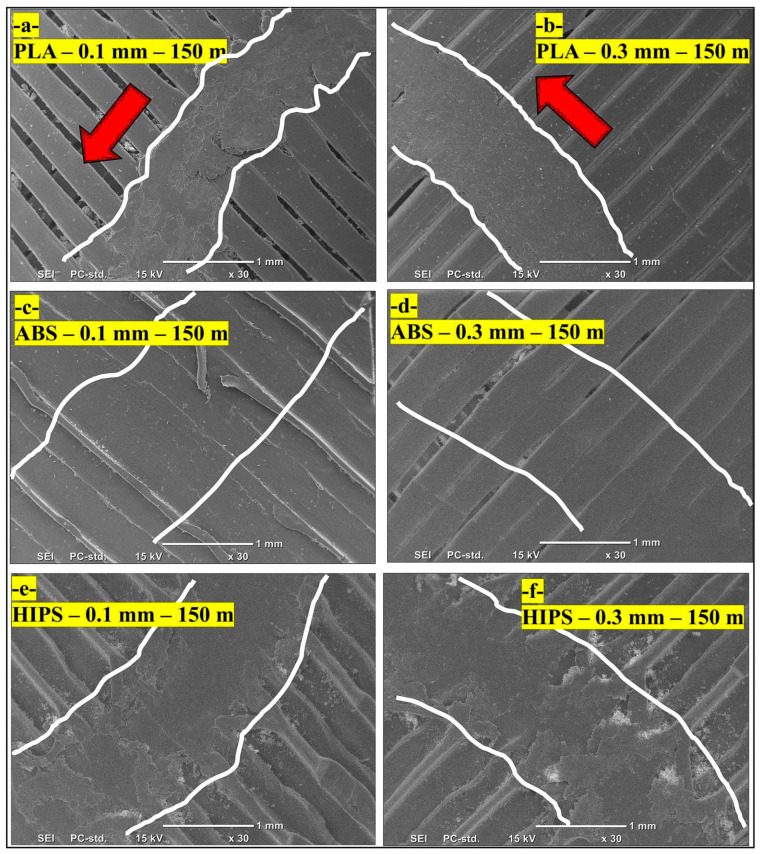
SEM images of the wear tracks of 3D-printed HIPS, PLA and ABS samples. (Red arrows imply sliding wear direction).

The wear rate results are presented in Figure 5. This quantity expresses the amount of wear per unit load and sliding distance; in other words, for better understanding, it expresses the change in wear speed depending on the load and friction time. When Figure 5 is examined in the light of this information, it can be seen that the wear rate results are similar to the wear volume results, varying with the material type and layer thickness. Post-test SEM imaging at 300 m revealed a continuous, compacted third-body film that filled surface asperities (Figure 6b,d), while profilometry showed a 25 ± 4% decrease in track depth relative to 150 m. Similar debris-compaction films have been reported to lower the coefficient of friction in FDM-printed PLA and ABS after an initial run-in stage rather than through any intrinsic self-repair of the polymer matrix [43,44]. Hanon et al. [44] examined the tribological characteristics of several 3D-printed filaments, focusing on wear behavior assessment and the distinction between static and dynamic friction coefficients. Their findings show that there is little variation in wear patterns and friction between the white ABS and PLA specimens. Gray PLA, on the other hand, varies significantly, with a notable increase in the average wear and friction coefficient. However, as the sliding distance increases, it can be seen that the wear rate does not increase like the wear volume; on the contrary, it decreases. This result shows that, even if the sliding distance increases after the wear mechanism has entered a steady-state regime, the material loss on the surface of the sample is not directly proportional to the increase in the sliding distance. In fact, the amount of wear occurring per unit distance, or in other words, per unit time, is decreasing [45]. The theoretical wear resistance for FDM-made plastic spur gears was evaluated for three different thermoplastic materials: PLA, ABS, and PETG. ABS exhibited the highest wear at identical loads and rotational speeds, PLA exhibited intermediate wear, and PETG exhibited the best wear. In comparison with PLA and ABS, PETG demonstrated higher wear resistance, as demonstrated by the theoretical wear depth estimates. These results imply that PETG is a more resilient material for gears fabricated using FDM in applications in which improved wear performance is required under comparable operating circumstances [46]. While PETG was not tested in this study, its inclusion demonstrates how material composition and structure influence tribological behavior, reinforcing the observed differences between ABS, PLA, and HIPS. This comparison highlights the broader applicability of layer thickness effects on polymer wear resistance, supporting our study’s conclusions about parameter optimization for functional applications. As stated in ref. [47], unreinforced PLA exhibits the lowest wear resistance, as evidenced by the wear depth and width of the worn surface. On the other hand, PLA reinforced with 30% biocarbon exhibited the highest wear resistance. Wear volume analysis verified that total wear resistance during cyclic sliding against an alumina ball was greatly improved by biocarbon reinforcement. Nevertheless, embrittlement and microcracking are seen as biocarbon content rises; therefore, biocarbon probably helps improve wear resistance but questions remain regarding possible material instability under high reinforcement levels. According to ref. [48], experimental samples were prepared using ABS and PLA thermoplastics via a Stratasys F170 3D printer, with the key printing parameters investigated including the material deposition layer thickness, infill angle, infill pattern, and deposition orientation. A change in the thickness of the layer influenced both distortion and bonding, thereby affecting the wear rate of the material. The observed increased infill density shows that the actual wear rate increases because the distortion effects dominate over the improvements in bonding. A double-infill pattern results in a wear rate that is reduced with respect to a single-infill pattern because of an increase in material strength due to the enhanced structural integrity of the double infill.

### 3.3. Wear Track SEM Analysis

PLA exhibits moderate resistance to wear due to its brittle nature. Wear tracks and wear debris are clearly noticeable in the SEM images of all samples, especially for the 0.3 mm layer thicknesses (Figure 6) and for larger sliding distances. The increased layer thickness considerably increased the amount of wear, which resulted from the brittle nature of the PLA material. The brittleness of the material initiates cracking and propagation, which causes material loss between layers. Although thinner layers indeed show reduced wear (0.1 mm—Figure 6a), they still show plastic deformation. Poor interlayer bonding in thicker layers reduces the wear resistance of PLA, making it more susceptible to damage under sliding conditions. The SEM study detects distinctive interlayer adhesion behavior of the material. PLA exhibits moderate adhesion with intermittent microcracks in accordance with its brittle fracture behavior. Among the three tested materials, ABS exhibited the best wear resistance, as evidenced by its much smoother wear tracks and very minimal observed wear debris in the SEM images. The 0.1 mm layer thickness (Figure 6c) shows consistent performance with very few material losses, indicating that ABS exhibits toughness characterized by high resistance to impact. However, instead of material removal during wear, ABS undergoes plastic deformation; hence, it absorbs the sliding energy and does not show significant surface damage (Figure 6d). Therefore, ABS is an excellent choice for applications requiring high wear resistance, particularly when thinner layers provide smoother surfaces with strong interlayer bonding. Among these three materials, HIPS exhibits the highest wear rate. From SEM images, one can see broad and deep tracks and heavy debris on the surface, especially for the 0.3 mm layer thickness samples (Figure 6f. In the case of HIPS, both plastic deformation (Figure 6e) and material removal are wear mechanisms, which become more severe when the layer thickness increases. This can be attributed to poor interlayer adhesion and the generation of internal voids in the structure; hence, its wear is characterized by increased material loss during sliding. Notwithstanding its high resistance to impact, HIPS exhibits poor wear resistance, especially for more massive layers; poor interlayer bonding enhances wear damage there. Distinct interfacial voids between layers correlate with observed debris accumulation in wear tracks, demonstrating weak interlayer cohesion. In contrast, ABS (Figure 6c,d) shows continuous material flow between layers, explaining its superior adhesion.

**Figure 7:**
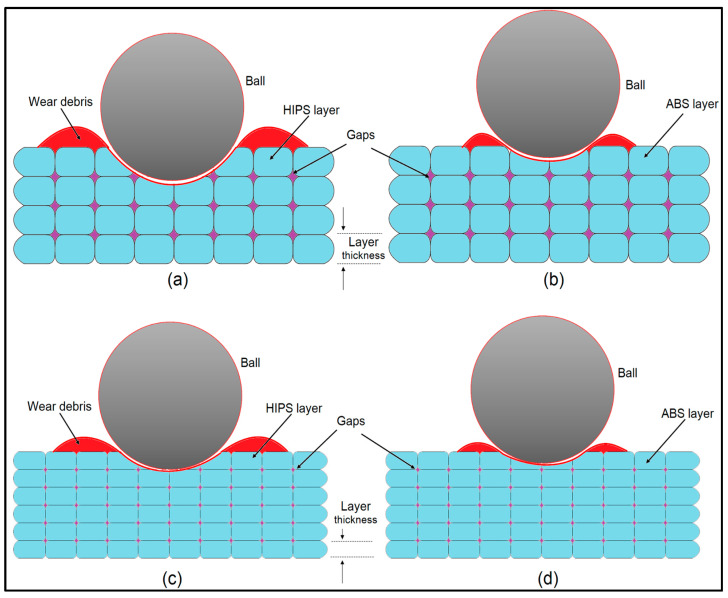
Schematic of wear debris formation and surface interaction in ball-on-disc testing: (**a**,**c**) HIPS layer, and (**b**,**d**) ABS layer.

The wear processes for ABS and HIPS differ significantly with regard to structural characteristics and printing settings for ABS and HIPS materials (Figure 7). The images show a comparison of the wear characteristics of both materials according to the layer thickness, voids, wear debris, and contact of the ball with the surface. This process is described below with the structural features of the materials: In Figure 7a,c, noticeably thicker layer structures composed of HIPS material are evident. HIPS is a material characterized by its strong impact resistance and low density; nevertheless, inadequate adhesion between layers and the resulting voids reduce wear resistance. The styrene and rubber ingredients in the HIPS composition enhance impact resistance, but may reduce the wear performance. In Figure 7b,d, thin layers formed with ABS are observed. ABS stands out owing to its amorphous structure and high strength. ABS, which consists of acrylonitrile, butadiene, and styrene, provides good layer adhesion and minimizes material loss during wear by plastic deformation. Owing to the thin layers, fewer gaps are formed, which contributes to ABS’s resistance to wear. In Figure 7a,c, more wear debris is seen formed during contact of the HIPS material with the ball. Although the thermoplastic elastomeric structure of HIPS exhibits high impact resistance, it causes these elastomeric regions to wear easily and causes material loss during wear. The main reason for the low wear resistance of HIPS is the weak bonding between the layers and the transformation of structural elastic regions into wear debris. Considering the ABS material, Figure 7b,d show less material debris during wear. This is because the amorphous and hard structure of ABS exhibits energy-absorbing properties and protects the material from plastic deformation during wear. The harder structure of ABS makes it resistant to wear and minimizes material loss. Therefore, the extrusion temperature directly affects the adhesion quality between the layers of both materials. The bonds between layers are weakened during wear due to the lower extrusion temperature of 255 °C used for HIPS, together with the formation of more voids. These voids account for the accumulation of wear debris displayed in Figure 7a,c. The elastomeric regions in the HIPS structure do not form strong bonds at low temperatures, thereby weakening its mechanical strength. The higher extrusion temperature (275 °C) used for ABS optimizes material flow and provides strong bonding between layers. This temperature resulted in fewer voids and wear debris, as shown in Figure 7b,d. The structural rigidity and amorphous structure of ABS provide better layer adhesion at high temperatures, which increases the wear resistance. The filling ratio and printing orientation play important roles in the wear resistance of materials. ABS offers a more powerful structure with high filling ratios and optimized printing orientations. Due to the amorphous structure, the delamination between layers is strong; hence, wear can cause minimal reductions in the amount of material. Severe wear is observed in HIPS for lower filling ratios or poor orientations because the layer adhesion is weak and elastic regions exist. This causes HIPS to create more voids and less resistance to wear. As a result, the wear mechanisms illustrated in Figure 7 are directly related to the structural properties and printing parameters of ABS and HIPS. ABS exhibits a more resistant performance against wear due to its amorphous and rigid structure, whereas the elastomeric structure and low bonding quality of HIPS lead to more material loss during wear.

### 3.4. Factorial Analysis

ANOVA analysis performed using the factorial method provides a powerful statistical tool to understand the interactive effects of multiple factors on an output; this method allows the determination of optimum parameters by distinguishing individual and joint variations in the factors, thus increasing the reliability of the experimental results. Table 1 shows the ANOVA results obtained using the factorial method for measuring wear volume. According to the ANOVA, the wear volume results show that the material used and printing parameters significantly affect the wear volume. The material factor has a dominant effect, explaining 59.76% of the total variance. This indicates that the variation created by different materials on wear volume is significantly high. The structural properties of 3D printing materials such as PLA, ABS, and HIPS have extensive effects on their respective wear properties. PLA normally possesses a structure that is hard and brittle, whereas ABS has been proven to be more flexible and resistant to impacts. HIPS is very resistant to impacts, and its processing abilities are high. The data in this table show that all these variations directly affected wear volume.

Layer thickness explains 21.32% of the total variance. Increasing the layer thickness generally results in lower resolution surfaces and thus more wear. Thinner layer thicknesses reduce the wear rate while producing smoother surfaces. Scratch length explains only 2.60% of the variance, indicating that this parameter has a relatively small effect on wear volume. However, despite its low contribution, scratch length plays a crucial role in wear behavior that cannot be completely neglected. In particular, longer scratches create more friction, which accelerates wear in some materials. Thick layer constructions and low bond strength form additional spaces on the surface in wear, which is the origin of wear debris. The relatively small contribution of scratch length to variation in wear volume (2.60%) can be accounted for by the attainment of steady-state wear conditions for long distances of sliding. Once initial surface asperities are levelled off and the contact interface achieves stability, the influence of additional scratch length on wear volume diminishes. In contrast, material type and layer thickness have direct effects on the intrinsic mechanical properties and interlayer adhesion of the printed test material specimens, respectively, which govern the initiation and development of wear. Furthermore, wear rate per unit distance is seen to diminish towards the end of sliding, which is indicative of the dominance of initial material properties over parameters dependent on distance. Therefore, even while scratch length increases exposure time to friction force, its contribution is relatively minimal compared to parameters governing the material’s own structural response.

When considering the two-way interactions, the interaction between material and layer thickness (13.34%) has a very significant effect. This indicates that each material responds differently to different layer thicknesses. For example, thicker layers can increase wear in flexible materials such as ABS, whereas this effect may be less in impact-resistant materials such as HIPS. Similarly, the interaction between material and scratch length (1.33%) indicates that the material structure affects the wear behavior together with the scratch length. As a result, the material used is the most important factor determining wear volume, followed by layer thickness and scratch length. The structural properties of the materials greatly affect the wear behavior, but the printing parameters modulate these behaviors.

The wear rate ANOVA results obtained from the factorial method are shown in Table 2. ANOVA using a factorial method was used to investigate the effects of different materials, layer thickness, and scratch length on the wear rate. It was deduced from the results of this study that the complete model contributed 100% to the wear rate. This indicates that the results of this research are quite significant. The materials (52.25%), layer thickness (20.10%), and scratch length (4.12%) significantly contributed to the wear rate. Therefore, the influence of material is greater. This can be easily demonstrated by examining different types of mechanical and structural properties for different types of materials. ABS is a tough material that is mainly impact-resistant, while PLA is a biodegradable material that is extremely brittle in nature. As might be expected, these features result in marked variations in wear resistance. HIPS is noted for excellent flexibility and exceptional shock resistance; the latter, in particular, is particularly sensitive to changes in the wear rate. The layer thickness also significantly affected the wear rate. Thinner layers increase wear resistance by providing a more homogeneous material structure, whereas thicker layers exhibit lower wear resistance due to voids in the microstructure and poor adhesion. Scratch length had a 4.12% effect. However, long scratch paths provide more wear on the surface, and this effect is much smaller than the material and layer thickness. When examining two-way and three-way interactions, the interaction between material and layer thickness contributed 13.34%. This shows that different layer thicknesses in different materials can significantly affect the wear rate. The interaction between the material and scratch length created a 4.00% effect. As a result, this analysis shows that the material type makes the greatest contribution to the wear rate, followed by the layer thickness and scratch length. In terms of optimizing the wear behavior, the mechanical properties and manufacturing parameters of materials are of great importance. ANOVA was used to identify the processing parameters with the most significant influence on wear rate. The results indicate that fill density has the greatest impact on wear rate, followed by extrusion temperature and nozzle speed. The fill density, extrusion temperature, and nozzle speed contributed to the wear rate at percentage contributions of 50.19%, 38.68%, and 10.38%, respectively. High fill density and extrusion temperatures coupled with appropriate nozzle speeds improve the bonding between the deposited layers, increasing the crystallinity of the material and thereby improving the wear resistance [49]. As stated in ref. [50], when an applied load and sliding speed are applied to FDM-manufactured parts, the build orientation has a substantial impact on both the wear behavior and the friction coefficient. Building at a 45° angle, applying little weight, and sliding at a modest speed—all of which reduce the friction coefficient—are the keys to achieving optimal performance. The results indicate that medium orientation angles, higher velocities, and reduced loads are best for consistent wear. These findings indicate that orientation plays an important role in enhancing FDM parts because it affects surface features, such as friction and wear, and mechanical properties, such as strength and dimensional accuracy. Kumar et al. [51] focused on finding the optimum process parameters for a Flashforge Dreamer NX (Single Extruder) 3D printer to fabricate test specimens with minimum wear rates. From the ANOVA results presented in Table 2 it can be seen that among the three identified input variables (layer thickness, infill density, and print speed), layer thickness and infill density are more significant variables than the other two, regarding variations in the wear rate under the present experimental conditions. This implies that readjustments of the two parameters are more important than modifying the print speed to reduce the wear rate, which provides useful insights into optimizing 3D-printed parts for durability and better performance. Mourya et al. [52] investigated the influence of printing parameters on the friction and wear properties of 3D-printed textured samples fabricated through FDM using ABS and PLA. The layer thickness, nozzle temperature, line width, and printing speed were the main factors affecting FDM. The experimental results showed that line width was the factor that most affected the tribological performance, followed by the layer thickness and printing speed. The printing settings for ABS and PLA were optimized via a gray relational analysis. When the layer thickness and nozzle temperature increase, the wear rate first decreases but then gradually increases. It increases linearly as the printing speed increases. Singh and Bharti [53] investigated the parametric effects of extruder temperature, print speed, infill density, and layer thickness on the wear behavior of PLA specimens fabricated via fused deposition modeling (FDM). The Taguchi L16 Orthogonal Array served as the basis for selecting the input parameter combinations. The minimum average percentage difference, progressing from experimental to predicted values, for the regression model was 1.94%, whereas for the ANN model, it was 5.04%. This implies that an optimal wear performance can be achieved if the layer thickness is maintained within the range of 0.28–0.34 mm. The layer thickness should be maintained between 0.28 and 0.34 mm for optimal wear performance. The infill density was set to 70–72%, the print speed was 125–175 mm/s, and the extruder temperature was set at 195–202 °C. In this work, mechanical property tests were conducted regarding five process variables in an FDM 3D-printed thermoplastic ASA polymer filament. The experiments were designed using Minitab Software of Version 21.4.0. Using the experimental design as a guide, an L18 orthogonal array was built to direct specimen creation. The results show that the two most important factors influencing the mechanical performance of 3D-printed ASA items are infill density and layer height. These results are essential for fine-tuning FDM fabrication to improve mechanical performance [54].

**Figure 8:**
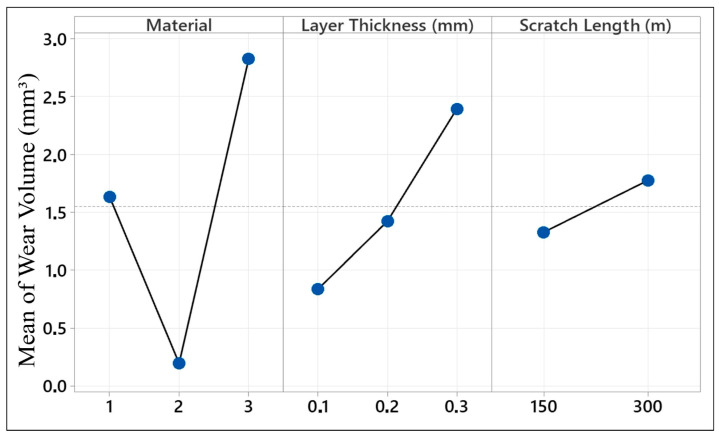
Mean effect plot for wear volume (PLA:1, ABS:2, and HIPS:3).

In the graph presented in Figure 8, the effects of different materials (PLA, ABS, and HIPS) produced with a 3D printer on wear volume are presented. The graph shows the effects of each material, layer thickness, and scratch length on the average wear volume (mm^3^). Regarding the material effect, PLA (1) and HIPS (3) showed high wear volume, whereas ABS (2) had the lowest volume. To exemplify, PLA had an average wear volume of 1.5 mm^3^, whereas ABS had a wear volume of almost zero. However, HIPS had a high wear volume at about 3 mm^3^. This could be explained by the fact that ABS outperformed the others because it has an amorphous structure, which is generally stronger and more resistant to wear. Materials like HIPS and PLA tend to have higher wear volumes because they are flexible and can absorb less energy.

When the layer thickness (0.1 mm, 0.2 mm, 0.3 mm) variable was examined, it was observed that the wear volume increased with increasing layer thickness. While the 0.1 mm layer thickness had the lowest wear volume, this value exceeded 2 mm^3^ for the 0.3 mm thickness samples. Thick layers can increase wear volume because they create more internal defects and weak adhesion areas in the material structure. Finally, a positive relationship was observed between scratch length (150 m and 300 m) and wear volume. The wear volume was 1.5 mm^3^ for 150 m of scratch length, and this value increased to 2.5 mm^3^ for 300 m of length. This indicates that an increase in the scratch length may cause more material loss on the contact surface. According to this analysis, HIPS is the least resistant material to wear, while ABS is the most resistant material. It was also observed that the layer thickness and scratch length increased. These results reveal that parameters such as material selection and layer thickness must be carefully optimized, especially during the production process. 

**Figure 9:**
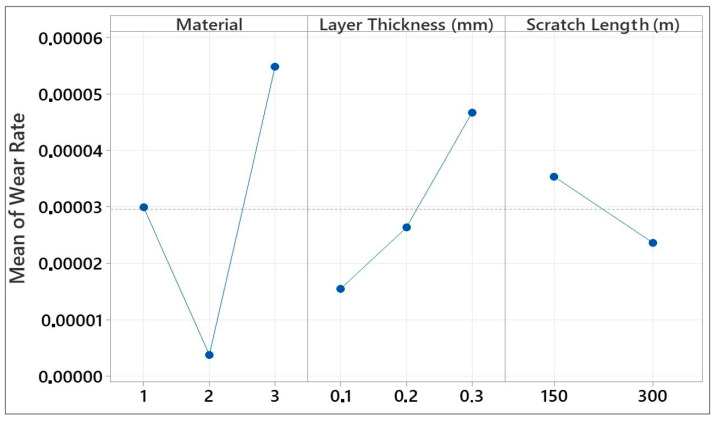
Mean effect plot for wear rate (PLA:1, ABS:2, and HIPS:3).

In the graph presented in Figure 9, we present the effects of materials produced with 3D printers on wear rate. Factors such as material type, layer thickness, and scratch length were evaluated on the basis of the wear rate. Examining the effect of material, PLA and HIPS showed high wear rates, whereas ABS showed the lowest wear rate. While PLA’s wear rate was around 0.0003 mm^3^/Nm, the rate for ABS was almost zero. For HIPS, the wear rate was about 0.0005 mm^3^/Nm. In Figure 8, the case for ABS’s wear volume being ‘almost zero’ is presented by the quantitative result of wear testing, where the observed wear volume for ABS for 0.1 mm layer thickness and 150 m scratch length was 0.05 mm^3^. Even for higher layer thicknesses and higher scratch lengths, ABS specimens had wear volumes of less than 0.15 mm^3^, which is considerably less than PLA (1.5 mm^3^) and HIPS (3 mm^3^) under comparable conditions. ABS’s excellent performance is the result of the amorphous character of ABS and superior interlayer adhesion, which minimizes plastic deformation and material disfigurement under the process of sliding wear. ABS has higher resistance to wear due to its higher tensile strength. On the other hand, PLA and HIPS exhibited high wear rates because of their rigid but brittle structures.

It was observed that there was a positive correlation between layer thickness and wear rate. The lowest wear rate was observed at a 0.1 mm layer thickness, and the wear rate reached a maximum level at a 0.3 mm thickness. Thick layers are more susceptible to wear because they accumulate more material and internal errors during printing. In contrast, scratch length had a negative effect. Increasing the scratch length from 150 to 300 m reduced the wear rate. Therefore, a temperature rise in the material along the scratch and run-in smoothing abilities can be effective. In addition, long scratches may subject the material to less stress, potentially leading to a low wear rate. As a result, it can be seen that ABS exhibits the highest wear resistance, whereas HIPS exhibits the lowest wear resistance. While increasing the layer thickness increased the wear rate, increasing the scratch length reduced it. This analysis indicates that optimizing the material and printing parameters is crucial and that it is important to consider wear performance when selecting materials.

## 4. Conclusions

The research problem investigated in this study was to find the effect of material type, layer thickness, and scratch length on the wear behavior of 3D-printed polymers. The results showed that, among the considered factors, material type was the most significant factor affecting wear. Among the materials, ABS showed the highest wear resistance, followed by PLA and HIPS. ABS was much better because of its amorphous and durable structure, which exhibited the smallest material loss of all three polymers; for example, at 0.1 mm layer thickness and a 150 m sliding distance, the mean wear volume was only 0.05 mm^3^, and even under the harshest condition tested (0.3 mm layer thickness, 300 m), the value remained below 0.15 mm^3^. Regarding the layer thickness, an increase in the layer thickness led to an increase in the volume of wear for all tested materials. For example, the wear volume of the 0.1 mm layers were significantly lower than that of the 0.3 mm layers. This could be attributed to the higher probability of defects and voids in thicker layers, which compromise the adhesion between layers and enhance the susceptibility to wear. Specifically, increasing the thickness from 0.1 to 0.3 mm increased the wear volume by more than two times. The study also highlighted an unexpected link between scratch length and wear rate. From 150 m to 300 m, an increase in sliding distance lowered the wear rate coefficient by 27–40%, a trend we attribute to classical run-in smoothing: compacted debris forms a thin transfer film that redistributes contact stresses and shields the underlying polymer. No autonomous in situ healing of the bulk material is implied. However, scratch length was of relatively minor importance for the wear behavior, accounting for only 2.6%, while material type and layer thickness were far more influential. Further, the interaction between material and layer thickness accounted for 13.34% of the wear variance. This aspect is really important and highlights that, in applications in which wear resistance is critical, both material and printing parameters are critical. For example, ABS with a thinner layer exhibits superior wear resistance and thus could be recommended for high-wear applications like gears or mechanical components. In this respect, the presented research contributes much to the knowledge about wear behavior in 3D-printed materials, especially with respect to the optimization of printing parameters. The results indicate that with appropriate material and thickness choices, durability and performance in 3D-printed parts can be enhanced. Further investigation could reveal long-term wear under various environmental conditions.

## Figures and Tables

**Table 1:** ANOVA was performed using the factorial method for measuring wear volume.

Source	DF	Seq SS	Contribution	Adj SS	Adj MS
Model	17	34.7736	100.00%	34.7736	2.0455
Linear	5	29.0986	83.68%	29.0986	5.8197
Material	2	20.7803	59.76%	20.7803	10.3902
Layer Thickness (mm)	2	7.4133	21.32%	7.4133	3.7067
Scratch Length (m)	1	0.905	2.60%	0.905	0.905
Two-Way Interactions	8	5.1777	14.89%	5.1777	0.6472
Material × Layer Thickness (mm)	4	4.639	13.34%	4.639	1.1597
Material × Scratch Length (m)	2	0.4629	1.33%	0.4629	0.2315
Layer Thickness (mm) × Scratch Length (m)	2	0.0758	0.22%	0.0758	0.0379
Three-Way Interactions	4	0.4973	1.43%	0.4973	0.1243
Material × Layer Thickness (mm) × Scratch Length (m)	4	0.4973	1.43%	0.4973	0.1243
Total	17	34.7736	100.00%		

**Table 2:** ANOVA was performed using the factorial method for the wear rate.

Source	DF	Seq SS	Contribution	Adj SS	Adj MS
Model	17	0.000000015	100.00%	0.000000015	0.000000001
Linear	5	0.000000011	76.48%	0.000000011	0.000000002
Material	2	0.000000008	52.25%	0.000000008	0.000000004
Layer Thickness (mm)	2	0.000000003	20.10%	0.000000003	0.000000002
Scratch Length (m)	1	0.000000001	4.12%	0.000000001	0.000000001
Two-Way Interactions	8	0.000000003	20.01%	0.000000003	0
Material × Layer Thickness (mm)	4	0.000000002	13.34%	0.000000002	0
Material × Scratch Length (m)	2	0.000000001	4.00%	0.000000001	0
Layer Thickness (mm) × Scratch Length (m)	2	0	2.67%	0	0
Three-Way Interactions	4	0.000000001	3.51%	0.000000001	0
Material × Layer Thickness (mm) × Scratch Length (m)	4	0.000000001	3.51%	0.000000001	0
Total	17	0.000000015	100.00%		

## Data Availability

The datasets presented in this article are not readily available because the data are part of an ongoing study. Requests to access the datasets should be directed to the corresponding author.

## Abbreviations

The following abbreviations are used in this manuscript:

ABS	Acrylonitrile butadiene styrene
PLA	Polylactic acid
HIPS	High-impact polystyrene
ANOVA	Analysis of Variance
SEM	Scanning electron microscopy
